# Association of Estrogen Receptor 1 and Tumor Necrosis Factor *α* Polymorphisms with Temporomandibular Joint Anterior Disc Displacement without Reduction

**DOI:** 10.1155/2020/6351817

**Published:** 2020-10-12

**Authors:** Bartosz Dalewski, Agata Kamińska, Katarzyna Białkowska, Anna Jakubowska, Ewa Sobolewska

**Affiliations:** ^1^Department of Dental Prosthetics, Pomeranian Medical University, Szczecin, Poland; ^2^Department of Genetics and Pathology, Pomeranian Medical University, Szczecin, Poland; ^3^Department of Genetics and Pathology; and Independent Laboratory of Molecular Biology and Genetic Diagnostics, Pomeranian Medical University, Szczecin, Poland

## Abstract

**Objectives:**

The aim of this study was to investigate the role of *ESR1* rs1643821 and TNF-*α* rs1800629 as potential genetic factors regulating anterior disc displacement without reduction-mediated inflammatory pathway.

**Background:**

The temporomandibular joint is a complex synovial joint that allows mandibular movement in three directions. Although temporomandibular disorders are widespread, limited data is available on the biochemical characteristics of the displaced disc and quality of the surrounding soft tissue. Changes in degenerative tissue provoke disc displacement which involves secretion of inflammatory markers and sequential conversion of fibroblast-like cells into chondrocyte-like cells. Due to the high occurrence in female adolescents, the potential role of sex hormones in temporomandibular joint disorders has been speculated. Furthermore, anterior disc displacement without reduction severely affects the quality of life.

**Methods:**

124 Caucasian patients with a history of at least one anterior disc displacement without reduction within 3 months were enrolled. Anterior disc displacement without reduction was diagnosed based on clinical examination, diagnostic criteria (DC)/TMD, and cone-beam computed tomography/magnetic resonance imaging (CBCT/MRI). The control group consisted of 126 patients with no temporomandibular joint disorders. Genotyping of two single nucleotide polymorphisms, estrogen receptor 1 (*ESR1*) rs1643821, and tumor necrosis factor *α* (TNF-*α*) rs1800629 was performed.

**Results:**

*ESR1* rs1643821 showed significant *P* values (using chi-square analysis) revealing the difference in anterior disc displacement without reduction frequencies while TNF-*α* rs1800629 polymorphism was found to be statistically insignificant when compared to the control group. Furthermore, patients with a genotype of *ESR1* rs1643821 showed a decreased probability (OR = 0.412) against anterior disc displacement without reduction when compared to the GG genotype (OR = 1).

**Conclusion:**

*ESR1* rs1643821 with A allele frequency was lower in patients with anterior disc displacement without reduction compared to the control group. Thus, the rs1643821 variant is significantly associated with susceptibility to the anterior disc displacement without a reduction in European Caucasians. Conversely, TNF-*α* rs1800629 was a statistically insignificant factor against anterior disc displacement without reduction when compared to the control group.

## 1. Introduction

**Figure 1:**
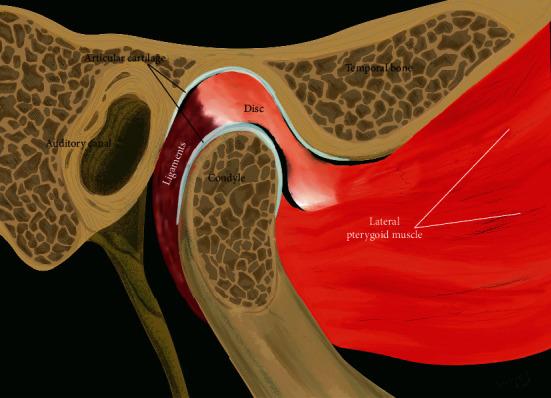
Normal TMJ closed.

**Figure 2:**
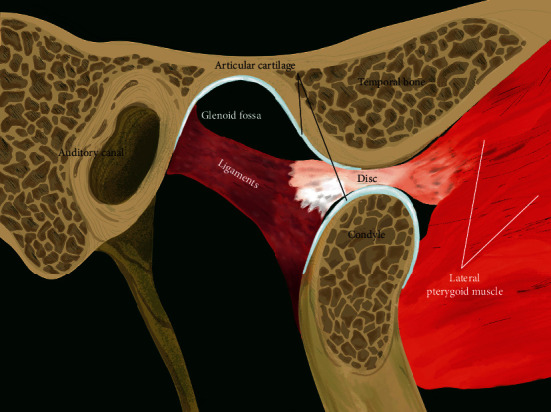
Normal TMJ open.

**Figure 3:**
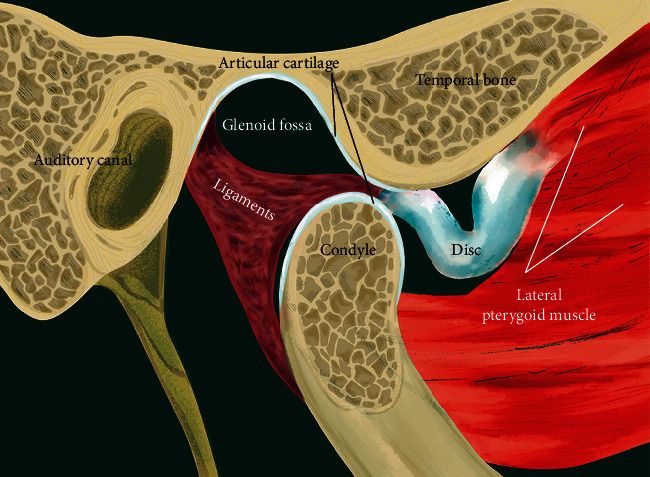
TMJ anterior disc displacement without reduction (ADDwoR).

**Figure 4:**
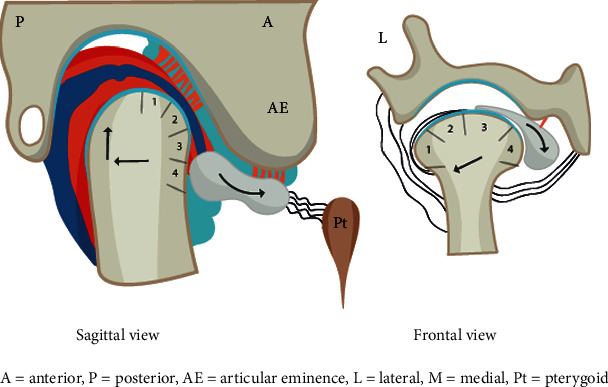
TMJ ADDwoR sagittal (a) and frontal view (b). Posterior and lateral ligaments become significantly overstretched and the disc gets distorted.

Temporomandibular joint (TMJ) is a complex synovial joint that allows mandibular movement in three directions. The three components of TMJ that enable such complex motion include mandibular condyle, glenoid fossa of the temporal bone, and fibrocartilaginous articular disc that is surrounded by synovial fluid (Figure 1). The prevalence of temporomandibular disorder (TMD-) related pain and severity has been reported to be twice in women than in men [1]. Accordingly, many previous studies have evaluated the potential role of gender on TMD pathogenesis by investigating sex hormones such as estrogen. Estrogen has been shown to play an important role in the symptomatology of female-predominant TMDs, synovitis, chondrocyte apoptosis, and inflammatory pain [2–5]. The most common arthropathy that causes dislocation of the disc-condyle complex is referred to as internal derangement (ID). Though its origin is not yet completely known, it is observed to be highly prevalent in female adolescents [6]. Furthermore, the most frequent type of TMJ ID is reported to be anterior disc displacement (ADD) with or without reduction (ADDwR or ADDwoR, respectively) [7]. In ADDwR, the disc slides out anteriorly from its native, functional position while the mandible opens and closes (Figure 2). On the other hand, in ADDwoR, the disc glides anteriorly and slightly medially to a lower resting position where it remains locked in the anterior joint recess (Figure 3). The displaced TMJ disc might be reducing at an earlier stage; however, is shown to later progress into a nonreducing form. The disc can also deform, torn, and elongate (Figure 4). Thus, if the disc fails to revert to its normal position, then the condylar movement disc becomes displaced and can prevent suitable condyle movement causing TMJ dysfunction [8]. Such medical condition severely affects the quality of life, and yet patients are required to undergo different treatment modalities and intensive rehabilitation for a prolonged time. However, approximately 15% of the patients develop a chronic form of TMJ pain that does not resolve and alleviate with therapy. Such patients are reported to show aggravated physical, behavioral, and psychological TMJ pain-related symptoms [9]. It has been reported that unilateral ADDwoR in teenagers can lead to mandibular asymmetry [10]. Furthermore, it has been proved that with prolonged time, the severity of mandibular asymmetry intensifies and can require orthognathic surgery [11]. Both, qualitative as well as quantitative condylar displacements are associated with TMJ DD. Osseous changes in the mandibular condyle are significantly influenced by TMJ DD, and its severity increases with TMJ DD progression [12]. On further development of ADDwoR, it can cause severe bone resorption which is known as idiopathic condylar resorption (ICR). ICR is a well-documented yet poorly diagnosed disease. Histologically, ADD is associated with alterations in the degenerative tissue that involves cellular repair mechanism where fibroblast-like cells phenotypically change into fibrochondrocytes and eventually into chondrocyte-like cells [13]. Although TMJ ID is quite common, limited data is available on the biochemical characteristics of the displaced disc and on the quality of the surrounding soft tissue. Nevertheless, the ligaments of the TMJ posterior band have been proved to play a significant role by preventing the displacement of the TMJ disc [14]. Due to the high occurrence of ICR in female adolescent patients, the role of sex hormones has been suggested in the ICR development. The evaluation of serum estrogen levels in patients with ICR has revealed a significant reduction in 17*β*-estradiol expression levels. It remains unclear and controversial whether it is the ADD or low serum estrogen expression that plays a significant role in ICR progression [15]. Subsequently, few studies have validated the influence of low serum estrogen level and ADDwoR on the mandible length and asymmetry and determined the potential factor that provokes ICR [16]. Although many questions still remain unanswered, there has been growing evidence on the ability of estrogens to modulate metabolism-related joint inflammation. Steroid hormones, specifically estrogen, act on the periphery and central nervous system (CNS) through their estrogen receptors (ER) (ER*α* and ER*β*) to mediate inflammation and central pain pathways [17]. For example, estrogen has been shown to directly act on monocytes and macrophages and regulate the production of proinflammatory cytokines such as interleukin-1 (IL-1), IL-6, and tumor necrosis factor *α* (TNF-*α*) [18]. Cytokines, IL-1*β*, and IL-6 have been detected in TMJ synovium during inflammation while IL-1 and TNF-*α* have been shown to inhibit proteoglycan synthesis and promote cartilage reabsorption as well as inflammation of joint structures and adjacent connective tissue [19]. ER*α*, also known as NR3A1, is one of the two main types of estrogen-activated receptors. In humans, ER*α* is encoded by *ESR1* and has been previously shown to be associated with distressed TMDs [13]. TNF-*α* is located on chromosome 6:31,575,567-31,578,336. rs1800629 is a single nucleotide polymorphism (SNP) in the TNF-*α*, also known as *TNFA-308*. rs1800629(A) allele is often referred to as 308.2 or TNF2 while the conventional G allele is labelled as 308.1 or TNF1. Furthermore, A allele is associated with upregulation in TNF-*α* expression (ensemble database). On the other hand, TNF-*β* promotes cell apoptosis and inflammatory responses as it binds to TNF receptor type 1 and 2, respectively. TNF-*β* is produced by lymphocytes, and its structure resembles TNF-*α*. TNF-*α* and TNF-*β* have been shown to have similar structure and functions such as 30% amino acid sequence similarity and identical widely distributed cellular receptors [20]. Consequently, it is assumed that TNF-*α* polymorphism affects the production and expression of proinflammatory cytokines (IL-1*α*, IL-1*β*, IL-6, TNF-*α*, and IFN*γ*) in TMD patients. Thus, we hypothesized that ESR1 and TNF-*α* may be expressed in patients with ADDwoR due to disruption in TMJ disc and adjacent tissue homeostasis.

In this study, we investigated the role of *ESR1* rs1643821 and TNF-*α* rs1800629 as potential genetic factors regulating the ADDwoR-mediated inflammatory pathway. We focused on Caucasian patients as selected SNPs have not been investigated in European Caucasians for their role in TMD.

## 2. Materials and Methods

### 2.1. Study Group

In this case-control study, we enrolled patients who were seeking treatment for TMD from 2014-2018 at the Department of Dental Prosthetics, Pomeranian Medical University, in Szczecin, Poland. Our study included 124 Caucasian patients from both the sexes. Patients with a history of ADDwoR within the last 3 months were included in the study. Prior informed formal consent was obtained from all patients enrolled in the study. ADDwoR was diagnosed based on clinical examination, diagnosed criteria for TMD (DC/TMD), and cone-beam computed tomography/magnetic resonance imaging (CBCT/MRI). The control group included 126 patients with no TMD based on DC/TMD analysis. Additional exclusion criteria for both the groups included the following:

1. Pathological tooth mobility (grade 1 or more using Miller index)
2. Have prior implemented occlusal splint therapy
3. Presence of occlusal support only in limited areas
4. Coexisting pathology or inflammation within jaws and muscles of the head and neck
5. Any concomitant metabolic diseases or known connective tissue disorders

### 2.2. SNP Selection

In this study, we investigated the role of *ESR1* rs1643821 and *TNF-α* rs1800629 as potential genetic factors mediating the signals of ADDwoR-induced inflammatory pathway. So far, the role of the selected SNPs has not been investigated in European Caucasians having TMD.

#### 2.2.1. DNA Isolation

Genomic DNA was extracted from oral epithelial cells using the SWAB Genomic Extraction GPB Mini Kit (Genoplast Biochemicals, Gdańsk, Poland) according to the manufacturer's instructions.

#### 2.2.2. Molecular Analyses

Genotyping of selected SNPs was performed by real-time PCR using TaqMan probes. SNPs, *ESR1* rs1643821 and TNF-*α* rs1800629, were analyzed using the predesigned Applied Biosystems TaqMan real-time PCR assays (Applied Biosystems, Foster City, CA, USA).

The reaction mix for each sample consisted of GoTaq® Probe qPCR Master Mix (Promega, Madison, WI, USA), TaqMan real-time PCR assays (Applied Biosystems, Foster City, CA, USA), and nuclease-free, deionized water, according to the manufacturer's instructions.

**Figure 5:**
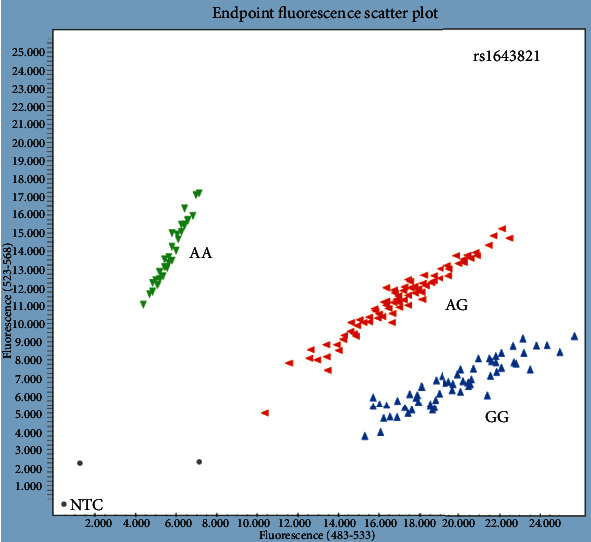
Allelic discrimination plot for ESR1 rs1643821.

**Figure 6:**
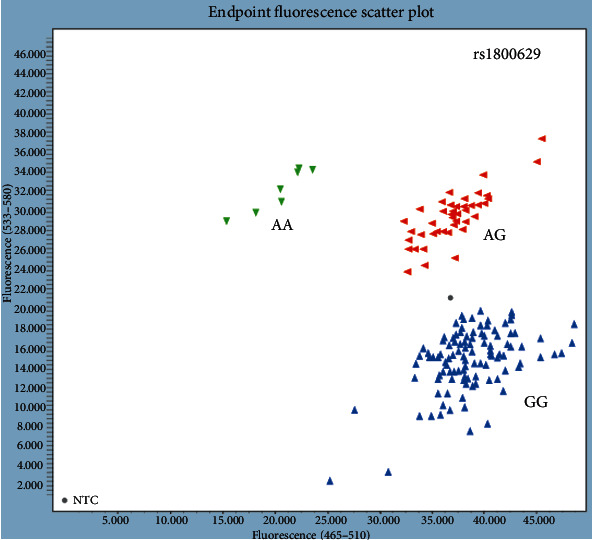
Allelic discrimination plot for TNF-*α* rs1800629.

Reaction mix, DNA, and no template control (NTC) were pipetted into 384-well plates (Axygen Inc., NY, USA). Real-time PCR was performed on LightCycler® 480 (Real-Time PCR System, Roche Diagnostics, Basel, Switzerland). Genotyping data was analyzed using the LightCycler480 Basic Software Version 1.5 (Roche Diagnostics, Basel, Switzerland). Allelic discrimination plots with the results of TaqMan genotyping for *ESR1* and TNF-*α* are shown in Figures 5 and 6.

### 2.3. Statistical Analysis

A significant difference in the distribution of genotypes was analyzed using Pearson's chi-square test. The logistic regression modelling was performed to analyze the influence of the investigated SNPs on ADDwoR. The data is presented as allele frequencies and odds ratio (OR) with 95% confidence interval (CI). The Mann–Whitney *U* test was performed to determine the age difference between the groups. *P* < 0.05 was considered to be statistically significant. The calculations were made using MATLAB (MathWorks Inc., US).

## 3. Results

### 3.1. Patient Characteristics

No significant genotypic differences were found with respect to age or sex between groups. The complete demographic information and clinical parameters of the study population are illustrated in Table 1. Experimental and clinical data were initially analyzed using descriptive statistics with respect to groups. Additionally, the chi-square test of independence was performed to validate the significant relationship between groups.

We did not find any significance based on the *P* value while comparing the genotypes of men and women that were enrolled in this case-control study. Thus, our study shows that gender has no influence on the frequency of TMJ ADDwoR.

On further analysis, polymorphism odds were evaluated with respect to most frequent allele combinations using 95% CI. The chi-square test at 5% confidence level was performed to determine the allele-based significant relationship between groups. The results of the OR analysis are shown in Table 2. In this study, the SNP marker, *ESR1* rs1643821, revealed a statistically significant difference (chi-square test, *P* = 0.014) in the frequency of developing TMJ ADDwoR. On the other hand, rs1800629 polymorphism in TNF-*α* was found to be statistically insignificant in comparison with the control group. Furthermore, patients with rs1643821 genotype AA showed a reduced probability (OR = 0.412) of developing TMJ ADDwoR in reference to genotype GG (OR = 1).

### 3.2. Data Verification by Logistic Regression Analysis

Furthermore, the unconditional logistic regression analysis was performed to validate the findings. Consistent with our earlier data in the study, we did not find any additional significance in the experimental data, except for *ESR1* rs1643821. None of the other statistical models showed any significance in the chi-square test vs. constant model.  Table 3 and Table 4 illustrate the results of the logistic regression analysis, respectively, for *ESR1* rs1643821 and TNF-*α* rs1800629.

The data suggested that a variation in the rs1643821 allele combination is a significant factor that increases the probability of developing TMJ ADDwoR.

## 4. Discussion

To the best of our knowledge, we are the first to investigate the role of *TNFA-308* (rs1800629) and *ESR1* rs1643821 in the pathogenesis of TMJ ADDwoR in European Caucasians. While we did not find significant changes in rs1800629 polymorphism, rs1643821 mutation with rare genotype AA was found to be lower in patients with ADDwoR compared to the control group. Furthermore, patients with rs1643821 genotype (AA+AG) showed a decreased probability (OR = 0.67) of developing ADDwoR compared to genotype GG allele carriers (OR = 1). Moreover, patients with genotype AA showed a decreased chance of developing TMJ ADDwoR. On the contrary to our findings, Furquim et al. have proved that *TNFA-308* rs1800629 polymorphism is positively associated with TMD occurrence in general, while in their study, Brazilian patients with TMD had 2.87 times increased probability of having GA genotype compared to the controls.

Furthermore, using the pressure pain threshold (PPT) test, they showed that rare A-allele homozygotes have decreased pain sensitivity for TMJ and anterior fascicle of the temporal muscle compared to the ancestral allele homozygotes [21]. Similarly, Yerliyurt et al. have shown that in the Turkish population, the TNF-*β* +252A/G variant was found to be significantly associated with susceptibility to TMD. However, they did not find a significant difference with respect to TNF-*β* +252A/G variant-related genotype (*P* = 0.010) or allele frequencies (*P* = 0.015) between the patient group and control group, respectively. A significant increase in TNF-*β* +252 AG genotype and G allele frequencies were observed in patients with TMD compared to healthy controls. The individuals with GG genotype and G allele were revealed to have an increased risk of developing TMD. Furthermore, a statistically significant association was observed when the patients were compared with the healthy controls based on the AA genotype vs. AG+GG genotypes (*P* = 0.002, OR = 2.23, 95%CI = 1.31 − 3.82). Additionally, the TNF-*β* +252A/G genotype distribution was shown to be associated with chewing problems (*P* = 0.046) [20]. TNF-*α* is a proinflammatory cytokine that plays a vital role in the pathogenesis of joint osteoarthritis (OA) [22]. A study conducted by Chen et al. has shown that IL-1*β* and TNF-*α* promote stiffness and impaired contractile function of articular chondrocytes [23]. Xue et al. investigated the TMJ OA mechanism in rats and reported that the female synovial membrane significantly upregulates the expression of proinflammatory factors. Further, they showed enhanced synovitis in female synovial membranes with severe cartilage degradation and bone deterioration as compared to the male synovial membrane during induced inflammation. Estrogen promotes TNF-*α*-induced mRNA expression of inducible nitric oxide synthase (iNOS), IL-1*β*, and monocyte chemoattractant protein 1 (MCP-1) in fibroblast-like synoviocytes. Furthermore, iNOS has been described as calcium-insensitive which may be due to its tight noncovalent interaction with calmodulin (CaM) and Ca^2+^.

Thus, tamoxifen (estrogen receptor antagonist) treatment can partially block TNF-*α*-induced upregulation of the proinflammatory cytokines. Furthermore, histomorphometrically, the osteoclast number and receptor activator of nuclear factor kappa-*Β* ligand (RANKL) expression surrounding the subchondral bone in the tamoxifen-treated rats were significantly reduced in patients compared to the osteoclast number and RANKL expression in the control group. Remarkably, no significant difference in the expression of ER*α* and ER*β* was detected in female and male synoviocytes by the real-time PCR and western blot analysis. These findings partly correlate with the data obtained in this study. However, there is a need for further investigation and confirmation in human trials [24]. This report evidently revealed that *ESR1* rs1643821 with rare A-allele in patients with ADDwoR was found at a lower frequency compared to that in the control group. Furthermore, the variation is significantly associated with susceptibility to ADDwoR in European Caucasians. In addition, in this case-control study, TNF-*α* rs1800629 polymorphism was found to be statistically insignificant compared to the control group.

### 4.1. Limitation of the Study

Further insight into other ethnic populations is now essential to validate the association of *ESR1* rs1643821 variant with susceptibility to ADDwoR worldwide. Plus, the role of estrogen sensitization and its mutual relationship with the biomarkers of inflammation in TMD patients requires additional consideration in larger cohorts.

## 5. Conclusion

To summarize, *ESR1* may be contributing to the pathogenesis of synovitis, and cartilage and bone degeneration in TMJ. This study is significant with regard to the pathogenesis of inflammatory joint diseases and provides a valuable reference for future investigations.

## Figures and Tables

**Table 1:** 

		Total *n* = 250		Case *n* = 124		Control *n* = 126		*P* ^∗^
		*N*	%	*N*	%	*N*	%	
Sex	F	200	80.00	104	83.87	96	76.19	0.129
	M	50	20.00	20	16.13	30	23.81	
Age (years)	<24	54	21.60	40	74.07	14	25.93	
	24-33	70	28.00	37	52.86	33	47.14	<0.001^∗∗^
	34-50	65	26.00	33	50.77	32	49.23	
	≥50	61	24.40	14	22.95	47	77.05	

^∗^Chi-square test, ^∗∗^Mann–Whitney *U* test.

**Table 2:** OR analysis.

SNP	Case	Control	OR	95% CI	*P* ^∗^
ESR1 rs1643821					
GG	44	28	1	-	-
AG	57	57	0.636	0.349-1.159	0.138
AA	22	34	0.412	0.201-0.842	0.014
TNF-*α* rs1800629					
GG	92	89	1	-	-
AG	26	33	0.901	0.422-1.376	0.367
AA	6	2	2.902	0.570-14.763	0.236

^∗^Chi-square test.

**Table 3:** Logistic regression modelling for ESR1 rs1643821.

	Estimate	SE	Wald test	*P*	OR	OR 95%	-OR 95%
Intercept ESR1 rs 1643821	0.452	0.242	3.496	0.0615	1.571	0.978	2.524
GG	0.000	-	-	-	1.000	-	-
AG	-0.452	0.306	2.184	0.1394	0.636	0.349	1.159
AA	-0.887	0.365	5.906	0.0151	0.412	0.201	0.842

**Table 4:** Logistic regression modelling for TNF-*α* rs1800629.

	Estimate	SE	Wald test	*P*	OR	OR 95%	-OR 95%
Intercept TNF-*α* rs1800629	0.033	0.149	0.050	0.8236	1.034	0.772	1.383
GG	0.000	-	-	-	1.000	-	-
AG	-0.272	0.301	0.812	0.3677	0.762	0.422	1.376
AA	-1.065	0.830	1.684	0.1992	2.902	0.571	14.763

## Data Availability

The data used to support the findings of this study have been deposited in the Department of Genetics and Pathology, Pomeranian Medical University, Szczecin, Poland.

## References

[1] Zhao Y. P., Ma X. C. (2006). Temporomandibular disorders related pain interaction with age, sex and imaging changes of osteoarthrosis. *Zhonghua Kou Qiang Yi Xue Za Zhi*.

[2] Wu Y. W., Bi Y. P., Kou X. X. (2010). 17-Beta-estradiol enhanced allodynia of inflammatory temporomandibular joint through upregulation of hippocampal TRPV1 in ovariectomized rats. *Journal of Neuroscience*.

[3] Wang X. D., Kou X. X., He D. Q. (2012). Progression of cartilage degradation, bone resorption and pain in rat temporomandibular joint osteoarthritis induced by injection of iodoacetate. *PLoS One*.

[4] Wang X. D., Kou X. X., Mao J. J., Gan Y. H., Zhou Y. H. (2012). Sustained inflammation induces degeneration of the temporomandibular joint. *Journal of Dental Research*.

[5] Wang X. D., Kou X. X., Meng Z. (2013). Estrogen aggravates iodoacetate-induced temporomandibular joint osteoarthritis. *Journal of Dental Research*.

[6] Kou X. X., Li C. S., He D. Q. (2015). Estradiol promotes M1-like macrophage activation through cadherin-11 to aggravate temporomandibular joint inflammation in rats. *Journal of Immunology*.

[7] Loreto C., Galanti C., Almeida L. E. (2012). Expression and localization of aquaporin-1 in temporomandibular joint disc with internal derangement. *Journal of Oral Pathology & Medicine*.

[8] Xie Q., Yang C., He D. (2016). Will unilateral temporomandibular joint anterior disc displacement in teenagers lead to asymmetry of condyle and mandible? A longitudinal study. *Journal of Cranio-Maxillofacial Surgery*.

[9] Xie Q., Yang C., He D., Cai X., Ma Z. (2015). Is mandibular asymmetry more frequent and severe with unilateral disc displacement?. *Journal of Cranio-Maxillofacial Surgery*.

[10] Ahn S.-J., Chang M.-S., Choi J.-H., Yang I.-H., An J.-S., Heo M.-S. (2018). Relationships between temporomandibular joint disk displacements and condylar volume. *Oral Surgery, Oral Medicine, Oral Pathology & Oral Radiology*.

[11] Okeson J. P. (2007). *Management of Temporomadibular Disorders and Occlusion*.

[12] Collins M., Raleigh S. M. (2009). Genetic risk factors for musculoskeletal soft tissue injuries. *Medicine and Sport Science*.

[13] Wolford L. M. (2017). Idiopathic condylar resorption of the temporomandibular joint in teenage girls (cheerleaders syndrome). *Baylor University Medical Center Proceedings*.

[14] Gunson M. J., Arnett G. W., Formby B., Falzone C., Mathur R., Alexander C. (2009). Oral contraceptive pill use and abnormal menstrual cycles in women with severe condylar resorption: a case for low serum 17beta-estradiol as a major factor in progressive condylar resorption. *American Journal of Orthodontics and Dentofacial Orthopedics*.

[15] Ralston S. H., Russell R. G., Gowen M. (1990). Estrogen inhibits release of tumor necrosis factor from peripheral blood mono-nuclear cells in postmenopausal women. *Journal of Bone and Mineral Research*.

[16] Shanker G., Sorci-Thomas M., Adams M. R. (1994). Estrogen modulates the expression of tumor necrosis factor alpha mRNA in phorbol ester-stimulated human monocytic THP-1 cells. *Lymphokine and Cytokine Research*.

[17] Pettipher E. R., Higgs G. A., Henderson B. (1986). Interleukin 1 induces leukocyte infiltration and cartilage proteoglycan degradation in the synovial joint. *Proceedings of the National Academy of Sciences of the United States of America*.

[18] Saklatvala J. (1986). Tumour necrosis factor *α* stimulates resorption and inhibits synthesis of proteoglycan in cartilage. *Nature*.

[19] Quinelato V., Bonato L. L., Vieira A. R., Granjeiro J. M., Tesch R., Casado P. L. (2018). Association between polymorphisms in the genes of estrogen receptors and the presence of temporomandibular disorders and chronic arthralgia. *Journal of Oral and Maxillofacial Surgery*.

[20] Yerliyurt K., Nursal A. F., Tekcan A., Karakus N., Tumer M. K., Yigit S. (2019). Effect of a functional variant of tumor necrosis factor-*β* gene in temporomandibular disorders: a pilot study. *Journal of Clinical Laboratory Analysis*.

[21] D'Aurea Furquim B., Flamengui L. M. S. P., Repeke C. E. P., Cavalla F., Garlet G. P., Conti P. C. R. (2016). Influence of TNF-*α*-308 G/A gene polymorphism on temporomandibular disorder. *American Journal of Orthodontics and Dentofacial Orthopedics*.

[22] El-Tahan R. R., Ghoneim A. M., El-Mashad N. (2016). TNF-*α* gene polymorphisms and expression. *Springerplus*.

[23] Chen C., Xie J., Rajappa R., Deng L., Fredberg J., Yang L. (2015). Interleukin-1 and tumor necrosis factor- increase stiffness and impair contractile function of articular chondrocytes. *Acta Biochimica et Biophysica Sinica*.

[24] Xue X.-T., Zhang T., Cui S.-J. (2018). Sexual dimorphism of estrogen-sensitized synoviocytes contributes to gender difference in temporomandibular joint osteoarthritis. *Oral Diseases*.

